# Autoimmune Disease Prevalence in a Multiple Sclerosis Cohort in Argentina

**DOI:** 10.1155/2014/828162

**Published:** 2014-08-06

**Authors:** Mauricio F. Farez, María E. Balbuena Aguirre, Francisco Varela, Alejandro A. Köhler, Jorge Correale

**Affiliations:** ^1^Department of Neurology, Institute for Neurological Research Dr. Raúl Carrea, FLENI, Montañeses 2325, 1428 Ciudad Autónoma de Buenos Aires, Argentina; ^2^Hospital de Clínicas José de San Martín, Avenida Córdoba 2351, 1428 Ciudad Autónoma de Buenos Aires, Argentina

## Abstract

*Background*. Comorbid autoimmune diseases in MS patients have been studied extensively with controversial results. Moreover, no such data exists for Latin-American MS patients. *Methods*. We conducted a case-control study aimed to establish the prevalence of autoimmune disorders in a cohort of Argentinean MS patients. *Results*. There were no significant differences in autoimmune disease prevalence in MS patients with respect to controls. The presence of one or more autoimmune disorders did not increase risk of MS (OR 0.85, 95% CI 0.6–1.3). *Discussion*. Our results indicate absence of increased comorbid autoimmune disease prevalence in MS patients, as well as of increased risk of MS in patients suffering from other autoimmune disorders.

## 1. Introduction

Multiple sclerosis (MS) is a complex autoimmune disease arising as a result of complex interactions between genetic and environmental factors [1]. The fact that it shares susceptibility genes with other autoimmune diseases raises the question as to whether MS cooccurs more frequently with these diseases than in the general population. Several studies addressing this question have been published with conflicting results. Some have shown increased prevalence of autoimmune diseases in MS patients [2, 3], whereas others claim those findings to reflect biases resulting from greater awareness and reporting of symptoms by MS patients [4, 5]. To the best of our knowledge, no studies have been published addressing autoimmune comorbidity rates in Latin-American MS patients.

## 2. Methods

We conducted a case-control study with relapsing remitting MS patients according to 2010 McDonald criteria [6]. Eligible patients were contacted via email and asked to complete a standardized questionnaire on recognized autoimmune or immune-mediated conditions (autoimmune thyroid disease, asthma, atopic dermatitis, type I diabetes, pernicious anemia, autoimmune uveitis, psoriasis, celiac disease, Crohn's disease, rheumatoid arthritis, Addison's disease, ulcerative colitis, and systemic lupus erythematosus; see Supplementary Material for Questionnaire available online at http://dx.doi.org/10.1155/2014/828162). Additional questions not relevant to the study were also included (quality of life, self-reported disease severity, weight and obesity, smoking habits, and relatives with MS) and patients were unaware of the hypothesis being tested. The control group comprised age- and gender-matched patients consulting the Pain Clinic at our center for headache and low back pain. Power calculations indicated that 199 MS patients and 199 controls would be necessary in order to demonstrate a twofold increase in the risk of autoimmune disease with *α* = 0.05 and *β* = 0.2. Differences in autoimmune disease presence between MS patients and controls were assessed using *χ* ^2^ test and Fisher's exact test. Risk of MS given the presence of any other autoimmune disease was assessed by logistic regression. Statistical analyses were performed using Stata v12.1. The study was approved by the Institutional Ethics Committee, and all subjects signed an informed consent form.

## 3. Results

Two hundred and fifty-five relapsing remitting MS patients were identified from the database and invited to participate, forty-four of whom refused or were unavailable, leaving two hundred and eleven eligible for analysis (Table 1). Twenty-eight percent of MS patients reported at least one autoimmune comorbidity, with no significant differences with respect to healthy controls (32%, *χ* ^2^ 0.45). Most frequently reported autoimmune conditions in MS patients were thyroid disease (9%), asthma (6%), atopic dermatitis (5%), and type I diabetes (3%). No differences between MS patients and controls were detected for any of the autoimmune disorders included (Table 2). Finally, presence of one or more autoimmune disorders did not increase risk of developing MS (OR 0.85, 95% CI 0.6–1.3) nor did age of diagnosis or gender show any effect on autoimmune disease rates.

## 4. Discussion

Our findings are in line with results published in a large cohort study conducted in Sweden [4], where increased risk of autoimmune disease prevalence was only noticeable after MS diagnosis, suggesting a surveillance bias. In contrast, several other publications recently reviewed in a meta-analysis have shown increased risk for autoimmune thyroid disorders, inflammatory bowel disease, and psoriasis [2]. In Caucasians, the HLA-DRB1∗1501 gene appears to confer most of the genetic susceptibility to MS [7]. Although Latin-American populations present singular genetic backgrounds, Argentinean MS patients show HLA class II gene frequencies similar to those observed in Caucasian populations [8]. In addition, genome-wide association studies have now revealed a large number of individual non-HLA genes modestly increasing MS risk [9]. The majority of these genes were not associated to other autoimmune disorders; however at least four genes have been linked to MS and other comorbid autoimmune disease [10]. The fact that no clear increase in cooccurrence has been found despite shared susceptibility gene presence highlights the complex interactions existing between genetic and environmental factors in autoimmune disorders.

## 5. Conclusions

In conclusion, our results indicate absence of increased comorbid autoimmune disease prevalence in MS patients, as well as of increased risk for developing MS in patients suffering from other autoimmune disorders.

## Supplementary Material

Questionnaire used to query the presence of autoimmune comorbidities in MS patients and controls.

## Figures and Tables

**Table 1:** Demographic and clinical features of MS patients and controls.

	MS patients (*n* = 211)	Controls (*n* = 211)
Age	40.4 ± 10	40.2 ± 10
Gender (women, *n*, %)	163 (77%)	163 (77%)
EDSS (median, range)	1 (0–8.5)	NA

**Table 2:** Comorbid autoimmune disease prevalence and risk of MS.

	MS, *n* (%)	Control, *n* (%)	OR (95% CI)	*P* value
Any autoimmune disease	60 (28%)	67 (32%)	0.85 (0.6–1.3)	0.5
Autoimmune thyroid disease	20 (9%)	13 (6%)	1.59 (0.8–3.3)	0.2
Asthma	11 (6%)	22 (11%)	0.5 (0.2–1)	0.06
Atopic dermatitis	10 (5%)	18 (9%)	0.54 (0.2–1.2)	0.1
Type I Diabetes	5 (3%)	5 (3%)	1 (0.3–3.4)	1
Pernicious anemia	3 (2%)	7 (4%)	0.45 (0.1–1.9)	0.3
Autoimmune uveitis	2 (1%)	1 (0.5%)	2.02 (0.2–22)	0.6
Psoriasis	1 (0.5%)	7 (4%)	0.13 (0–1.1)	0.06
Celiac disease	1 (0.5%)	3 (2%)	0.33 (0–3.2)	0.3
Crohn's disease	1 (0.5%)	1 (0.5%)	1 (0.1–16)	0.9
Rheumatoid arthritis	1 (0.5%)	3 (2%)	0.3 (0–3.2)	0.3
