# Supplementary material for: Autoimmune Disease Prevalence in a Multiple Sclerosis Cohort in Argentina
Source: Mult Scler Int. 2014 Aug 6;2014:828162. doi: 10.1155/2014/828162 (PMC4142150; doi:10.1155/2014/828162)
Supplement: Supplementary file 1 — Questionnaire used to query the presence of autoimmune comorbidities in MS patients and controls. [file 828162.f1.doc]

CUESTIONARIO SOBRE ENFERMEDAD TIROIDEA

1. ¿Alguna vez ha recibido tratamiento para enfermedad tiroidea en el pasado? *(Have you ever received treatment for any thyroid condition?*)

|  | Sí (Yes) |
| --- | --- |
|  | No (No) |

1. ¿Fue esta enfermedad llamada enfermedad de Graves? (*Was this condition called Graves Disease?*)

|  | Sí (Yes) |
| --- | --- |
|  | No (No) |

1. ¿Fue esta enfermedad llamada enfermedad de Hashimoto? (*Was this condition called Hashimoto disease?*)

|  | Sí (Yes) |
| --- | --- |
|  | No (No) |

1. ¿Fue esta enfermedad llamada hipertiroidismo? (*Was this condition called hyperthyroidism?*)

|  | Sí (Yes) |
| --- | --- |
|  | No (No) |

1. ¿Fue esta enfermedad llamada hipotiroidismo? (*Was this condition called hipothyroidism?*)

|  | Sí (Yes) |
| --- | --- |
|  | No (No) |

1. ¿Alguna vez tomó levotiroxina? (*Have you ever been prescribed Levothyroxine?*)

|  | Sí (Yes) |
| --- | --- |
|  | No (No) |

1. Si tomó ¿Qué dosis le dieron? (*If yes, do you remember the dosage?)*

1. ¿Alguna vez tomó propiltiouracilo? (*Have you ever been prescribed propylthiouracil?*)

|  | Sí (Yes) |
| --- | --- |
|  | No (No) |

1. Si tomó ¿Qué dosis le dieron? (*If yes, do you remember the dosage?)*

1. ¿Alguna vez tomó metimazol? (*Have you ever been prescribed methimazole?*)

|  | Sí (Yes) |
| --- | --- |
|  | No (No) |

1. Si tomó ¿Qué dosis le dieron? (*If yes, do you remember the dosage?)*

1. ¿Alguna vez recibió terapia con radiaciones para la tiroides? (*Have you ever received radiation therapy on your thyroides?)*

|  | Sí (Yes) |
| --- | --- |
|  | No (No) |

1. ¿Alguna vez le realizaron una cirugía de tiroides? (*Did you undergo thyroid surgery?)*

|  | Sí (Yes) |
| --- | --- |
|  | No (No) |

1. ¿Quién es su médico de cabecera y en que Institución médica trabaja? (*What is the contact information of your primary care physician?)*

1. ¿Tiene algún familiar con enfermedad tiroidea? (*Do you have any relatives with thyroid conditions?)*

|  | Sí (Yes) |
| --- | --- |
|  | No (No) |

1. Si tiene un familiar ¿Qué grado de parentesco tiene? *(If yes, can you specify which?)*

1. ¿A qué edad le diagnosticaron cualquiera de las anteriores? (*At what age where you diagnosed with thyroid conditions?)*

CUESTIONARIO SOBRE OTRAS ENFERMEDADES AUTOINMUNES

1. ¿Alguna vez le diagnosticaron Lupus, o Lupus Eritematoso sistémico Sí (Yes) No (No)

*(Have you ever been diagnosed with Lupus or systemic lupus erythematosus?)*

2. ¿Alguna vez le diagnosticaron Artritis Reumatoidea Sí (Yes) No (No)

*(Have you ever been diagnosed with Reumathoid Arthritis?)*

3. ¿Si tiene Artritis Reumatoidea: que tratamiento recibe/recibió?

*(If you did, what treatment did you received?)*

4. ¿Alguna vez fue diagnosticado con diabetes? Sí (Yes) No (No)

*(Have you ever been diagnosed with Diabetes?)*

5. ¿Si contestó que sí, a que edad fue diagnósticado?

*(If you did, at what age were you diagnosed?)*

6. ¿Qué tratamiento recibe/recibió para su diabetes?

*(If you did, what treatment did you received for your diabetes?)*

7. ¿Alguna vez fue diagnósticado con Asma? Sí (Yes) No (No)

*(Have you ever been diagnosed with Asthma?)*

8. ¿A qué edad se la diagnosticaron?

*(If you did, at what age were you diagnosed?)*

9. ¿Cuándo tuvo su última crisis asmática?

(*When did you have your last asthma exacerbation?)*

10. ¿Qué tratamiento recibe/recibió para su asma?

*(What treatment did you received for your asthma?)*

11. ¿Alguna vez fue diagnosticado con psoriasis? Sí (Yes) No (No)

*(Have you ever been diagnosed with psoriasis?)*

12. ¿A qué edad se la diagnosticaron?

*(If you did, at what age were you diagnosed?)*

13. ¿Qué tratamiento recibe/recibió para su psoriasis?

*(What treatment did you received for your psoriasis?)*

14. ¿Alguna vez fue diagnosticado con colitis ulcerosa? Sí (Yes) No (No)

*(Have you ever been diagnosed with Ulcerative Colitis?)*

15. ¿A qué edad se la diagnosticaron? ……

*(If you did, at what age were you diagnosed?)*

16. ¿Qué tratamiento recibe/recibió para su colitis ulcerosa?

*(What treatment did you received for your ulcerative colitis?)*

17. ¿Alguna vez fue diagnosticado con enfermedad de Crohn? Sí (Yes) No (No)

*(Have you ever been diagnosed with Crohn’s disease?)*

18. ¿A qué edad se la diagnosticaron?

*(If you did, at what age were you diagnosed?)*

19. ¿Qué tratamiento recibe/recibió para su enfermedad de Crohn?

*(What treatment did you received for your Crohn’s disease?)*

20. ¿Alguna vez fue diagnosticado con enfermedad celíaca? Sí (Yes) No (No)

*(Have you ever been diagnosed with Celiac disease?)*

21. ¿A qué edad se la diagnosticaron?

*(If you did, at what age were you diagnosed?)*

22. ¿Cumple con Dieta libre de Gluten? Sí (Yes) No (No)

(*Are you currently on a gluten-free diet?)*

23. ¿Alguna vez fue diagnosticado con anemia perniciosa? Sí (Yes) No (No)

*(Have you ever been diagnosed with Pernicious anemia?)*

24. ¿A qué edad se la diagnosticaron?

*(If you did, at what age were you diagnosed?)*

25. ¿Recibió vitamina B12 como tratamiento? Sí (Yes) No (No)

(*Did you received vitamin B12 as a treatment?)*

26. ¿Alguna vez fue diagnosticado con enfermedad de Addison? Sí (Yes) No (No)

*(Have you ever been diagnosed with Addison’s disease?)*

27. ¿A qué edad se la diagnosticaron?

*(If you did, at what age were you diagnosed?)*

28. ¿Qué tratamiento recibe/recibió para su enfermedad de Addison?

*(What treatment did you received for your Addison’s disease?)*

29. ¿Alguna vez fue diagnosticado con eccema o dermatitis atópica? Sí (Yes) No (No)

*(Have you ever been diagnosed with Eczema or atopic dermatitis?)*

30. ¿A qué edad se la diagnosticaron?

*(If you did, at what age were you diagnosed?)*

31. ¿Qué tratamiento recibe/recibió para su eccema o dermatitis atópica?

*(What treatment did you received for your eczema or atopic dermatitis?)*

32. ¿Alguna vez fue diagnosticado con uveitis autoinmune? Sí (Yes) No (No)

*(Have you ever been diagnosed with autoimmune uveitis?)*

33. ¿A qué edad se la diagnosticaron?

*(If you did, at what age were you diagnosed?)*

34. ¿Qué tratamiento recibe/recibió para su uveitis autoinmune?

*(What treatment did you received for your autoimmune uveitis?)*
